# Potentiometric Studies of the Complexation Properties of Selected Lanthanide Ions with Schiff Base Ligand

**DOI:** 10.3390/ijms262110379

**Published:** 2025-10-25

**Authors:** Julia Barańska, Katarzyna Koroniak-Szejn, Michał Zabiszak, Anita Grześkiewicz, Monika Skrobanska, Martyna Nowak, Renata Jastrzab, Małgorzata T. Kaczmarek

**Affiliations:** Faculty of Chemistry, Adam Mickiewicz University in Poznań, Uniwersytetu Poznańskiego 8, 61-614 Poznań, Poland; julia.baranska@amu.edu.pl (J.B.); katarzyna.koroniak@amu.edu.pl (K.K.-S.); zabiszakm@amu.edu.pl (M.Z.); anita.grzeskiewicz@amu.edu.pl (A.G.); monika.skrobanska@amu.edu.pl (M.S.); martynan@amu.edu.pl (M.N.); renatad@amu.edu.pl (R.J.)

**Keywords:** lanthanide complexes, salen, potentiometric studies, luminescence

## Abstract

The synthesis, characterization, and equilibrium studies of complexes of selected lanthanide ions Eu(III), Gd(III), and Tb(III) with the ligand 1,3-bis(3-bromo-5-chlorosalicylideneamino)-2-propanol (H_3_L) are reported. It was found that in the solid state, the complexes with the formulas [Eu(H_3_L)_2_(NO_3_)_3_], [Gd(H_3_L)_2_(NO_3_)_3_], and [Tb(H_3_L)_2_(NO_3_)_3_] are formed. In solution, complexes with stoichiometries of Ln(III):H_3_L 1:1 and 1:2 were obtained. The ligand H_3_L was isolated in crystalline form, and its molecular structure and conformation were determined by single-crystal X-ray diffraction analysis. The compounds were further characterized by elemental analysis, infrared spectroscopy, ^1^H NMR, ^13^C NMR techniques, and mass spectrometry (ESI), confirming the formation of the Schiff base group. Stability constants of the complexes in solution were determined using potentiometric titration, providing insights into the metal-ligand binding equilibria. In addition, the spectroscopic properties of the ligand and its lanthanide(III) ion complexes were investigated by UV-Vis spectroscopy, which confirmed ligand-to-metal charge transfer interactions, as well as by luminescence measurements. The luminescence studies revealed inefficient energy transfer in [Eu(H_3_L)_2_(NO_3_)_3_] complexes, while no transfer was observed in [Tb(H_3_L)_2_(NO_3_)_3_] systems at any pH value. This behavior is attributed to the large energy gap between the ligand triplet state and the lowest resonant levels of the studied lanthanide ions.

## 1. Introduction

Although Schiff base compounds are among the most extensively studied, they continue to garner significant research attention. A prominent subgroup of Schiff base compounds, referred to as the salen family, arises from the condensation of salicylic aldehyde derivatives with primary diamines. The term salen is derived from the substrates employed in the earliest synthesis of these compounds: salicylaldehyde and ethylenediamine. The interest in this type of compound is caused by both the broad spectrum of applications and the relative ease with which they can be synthesized [1,2,3,4,5,6,7,8]. Salen-type compounds are widely used in coordination and materials chemistry. Their unique properties result from the ability to form stable complexes with various transition metal ions. Complexes of metal ions with salen (e.g., Mn, Co, Cr, V, Cu, Fe) are used as catalysts in many organic reactions, including epoxidation of alkenes, oxidation of sulfides to sulfoxides, or polymerization of olefins [9]. These compounds can also serve as models of biological systems; for example, they imitate the active centers of many organometallic enzymes (e.g., oxidases, catalases, peroxidases). Research on them helps to understand the mechanisms of natural enzymes [10]. Due to their electrochemical and photochemical properties, salen-type complexes are being studied as components of chemical sensors, magnetic and luminescent materials, and organic semiconductors. They are also used in medicine and pharmacy because of their anticancer, antibacterial, and antioxidant properties [11,12].

Salen-type complexes with lanthanide ions (e.g., Eu, Tb, Sm, Dy) are used as light emitters due to the high luminescence of lanthanides. They are used in organic light-emitting diodes (OLEDs), fluorescent markers, and optical sensors (e.g., metal ion and biomarker detection) [13,14,15,16].

Lanthanide ions show significant differences compared to *d*-metal ions. Their complexing behavior is primarily regulated by the size of ions, charge density, and shielding of 4f orbitals. They are characterized by high coordination numbers due to their large ionic radii, most often 8 to 12 [17,18,19]. Lanthanides can form complex crystal lattices and metal–organic frameworks (MOFs) with salen precisely because of their high coordination numbers [20,21,22]. Such structures can be used in gas storage, separation, or in the design of magnetic materials. Eu(III) and Tb(III) complexes are being studied as luminescent contrast probes in bioimaging, e.g., for visualizing cancer cells [23]. According to Pearson’s HSAB principle, Ln(III) ions behave like hard Lewis acids and preferentially bind to hard donor atoms such as oxygen and nitrogen. Typical ligands include hydroxide compounds, carboxylates, phosphates, sulfates, and polyamino-carboxylates [24]. The spatial and conformational structure of ligands also plays a crucial role in determining their ability to form stable complexes. Even subtle variations in geometry, such as dihedral angles or the orientation of donor groups, can significantly influence coordination behavior and the stability of complexes. Recent studies have demonstrated that different molecular conformers may exhibit distinct binding affinities and spectroscopic properties due to variations in steric and electronic effects [25,26]. Therefore, understanding how molecular geometry governs complex formation is essential for interpreting the stability and optical properties of such systems.

**Figure 1:**
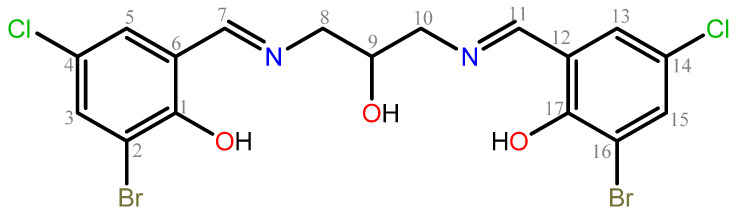
Chemical formula of 1,3-bis(3-bromo-5-chlorosalicylideneamino)-2-propanol, H_3_L.

Considering the properties of salen-type ligands and the chemical preferences of lanthanide(III) ions, we proposed a ligand in which the typical N_2_O_2_ set of donor atoms was changed to N_2_O_3_. In this paper, we presented the synthesis of 1,3-bis(3-bromo-5-chlorosalicylideneamino)-2-propanol, H_3_L (Figure 1), ligand, and its complexes with Eu(III), Gd(III), and Tb(III) ions. The application possibilities of the complexes are related to their stability in solutions, which is why the equilibrium constants and formation constants of the obtained compounds were studied.

## 2. Results and Discussion

The 1,3-bis(3-bromo-5-chlorosalicylideneamino)-2-propanol, H_3_L ligand was obtained by a condensation reaction between 3-bromo-5-chloro-2-hydroxybenzaldehyde and 2-hydroxy-1,3-propanediamine. The complexes of Eu(III), Gd(III), and Tb(III) ions were synthesized in a one-pot reaction between 3-bromo-5-chloro-2-hydroxybenzaldehyde and 2-hydroxy-1,3-propanediamine and the appropriate metal ion. As a result of the reactions carried out, mononuclear complexes [Eu(H_3_L)_2_(NO_3_)_3_], [Gd(H_3_L)_2_(NO_3_)_3_], and [Tb(H_3_L)_2_(NO_3_)_3_] were obtained. The compositions of the received compounds were determined based on single-crystal X-ray diffraction analysis, spectroscopic data (FTIR, UV-Vis, luminescence, mass spectrometric (ESI-MS), ^1^H and ^13^C NMR), and the stability constants of the complexes were defined in solution with the potentiometric method.

**Figure 2:**
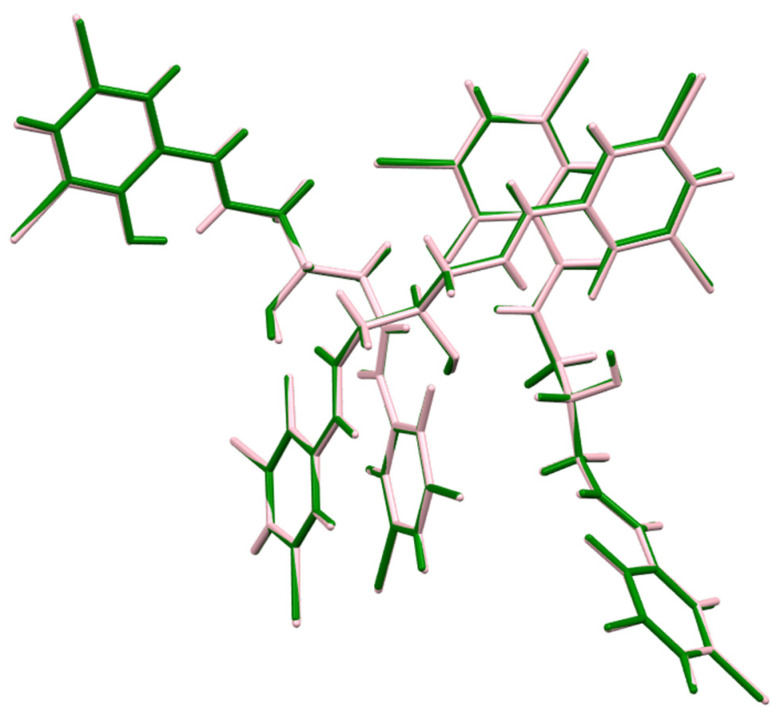
The molecular overlay of compound 1,3-bis(3-bromo-5-chlorosalicylideneamino)-2-propanol, H_3_L (green), and 1,3-bis(3,5-dibromosalicylideneamino)-2-propanol (pink).

The crystal structure of 1,3-bis(3-bromo-5-chlorosalicylideneamino)-2-propanol (Figure S1) is very similar to that of the one already published, 1,3-bis(3,5-dibromosalicylideneamino)-2-propanol [27]. In both cases, the asymmetric parts of the appropriate unit cells consist of 3 independent molecules, which occupy analogous positions (Figure 2). The unit cell similarity index equals 0.0088, and the isostructurality index [28] is 0.996, which proves an extremely high degree of isostructurality between those two derivatives.

**Figure 3:**
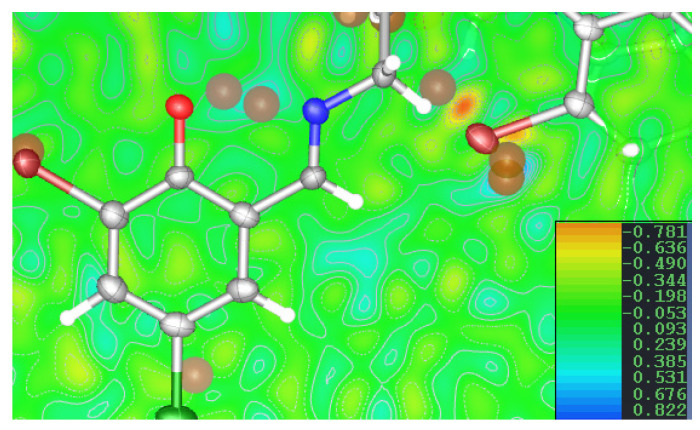
The contour map with the difference electron density map with q-peaks.

The only difference which has been found between those two compounds is the position of one certain, however important, hydrogen atom involved in the N32…O35 intermolecular hydrogen bond. It has to be admitted that establishing the position of the hydrogen atom by means of X-ray diffraction data may be quite a tricky issue. In the structure of H_3_L, some indications have been found, however, which implicate the possibility of a disordered hydrogen atom between atoms O35 and N32. The elongation of the C35-O35 bond (in comparison to the unprotonated oxygen atoms observed in the structure) indicates the possibility of protonation on the O34 atom; on the other hand, the C33-N32-C31 angle, of 121.54(4)°, also shows the N32 atom as a possible protonation center. (Table S1) The difference electron density map (Figure 3) shows the missing electron density concentrations, which can be connected with the hydrogen atom in question close to both O35 and N32 atoms, and two electron density maxima, of similar height (0.44/0.4 e). Therefore, it has been decided to model a disordered hydrogen atom; however, it should be noted that refining the shared hydrogen atom (at one position) yields the same refinement parameters.

Quite a detailed structural analysis of the molecular structure of 1,3-bis(3,5-dibromosalicylideneamino)-2-propanol has been described earlier [27], so, due to the almost perfect isostructurality, this discussion will not be repeated here. The crystal packing in 1,3-bis(3-bromo-5-chlorosalicylideneamino)-2-propanol is mostly determined by quite strong O-H··· hydrogen bonds and, to some extent, also by the stacking interactions between substituted aromatic rings. Additionally, there are weak C-H···Br and C-H···π hydrogen-bond-like interactions, which may also influence the arrangement of molecules in the crystal lattice. The geometry of selected hydrogen bonds has been summarized in Table 1, Table S2; the others can be found in the Supplementary Materials, together with bond distances, valence angles, and torsion angles for all three molecules of 1,3-bis(3-bromo-5-chlorosalicylideneamino)-2-propanol (Figure S2).

### 2.1. FT-IR Spectroscopy

FTIR studies were performed on the ligand H_3_L and the solid-state complexes [Eu(H_3_L)_2_(NO_3_)_3_], [Gd(H_3_L)_2_(NO_3_)_3_], [Tb(H_3_L)_2_(NO_3_)_3_] to investigate the coordination sphere in the obtained complexes. The spectra were recorded in the range of 4000–400 cm^−1^. The spectra of the complexes were compared to that of the ligand. FTIR spectroscopic analysis of the H_3_L ligand revealed the presence of bands with a wavelength of 1637 cm^−1^, which are from imine bonds formed as a result of the condensation reaction of amine and carbonyl groups. Comparing the position of this band in the spectra of the ligand and the complexes, a very small shift in this band was found: 1639 cm^−1^ (for Eu^3+^), 1640 cm^−1^ (for Gd^3+^), and 1640 cm^−1^ (for Tb^3+^), which may indicate that the azomethine nitrogen atoms do not participate in coordination with the metal ions. However, the decrease in the frequency of stretching of the C=N bond due to the reduction in the double bond character is usually attributed to the participation of the nitrogen atom in the coordination with the metal ion. Data collected from both X-ray crystallography and spectroscopy reveal that in the ligand, occurs a proton transfer from hydroxyl groups to nitrogen atoms, which causes the C=N double bond to elongate. Therefore, when the metal ion coordinates to the donor nitrogen atom, we observe a very small shift in C=N band. Additionally, FTIR spectra of the ligand and complexes present a broad band in the range 3254–3064 cm^−1^ attributed to the stretching vibration of OH groups. The FTIR spectra of the ligand and complexes show bands at 1133 cm^−1^ for the ligand and at 1142 cm^−1^ for the [Eu(H_3_L)_2_(NO_3_)_3_], 1146 cm^−1^ for [Gd(H_3_L)_2_(NO_3_)_3_], and 1440 cm^−1^ for [Tb(H_3_L)_2_(NO_3_)_3_] complexes attributed to the C-O bond. The shift in the band confirmed coordination of lanthanide ions to donor oxygen atoms. The FTIR spectra of the complexes exhibited bands in the ranges 1437–1206 cm^−1^ for Eu complex, 1444–1209 cm^−1^ for Gd complex, and 1440–1208 cm^−1^ for Tb complex characteristic of coordinated nitrate groups. The difference between these bands of about 233 cm^−1^ suggests that the nitrate counterions are coordinated in a bidentate fashion.

### 2.2. Equilibrium Study

The research involved the examination of the acid–base properties of 1,3-bis(3-bromo-5-chlorosalicylideneamino)-2-propanol (H_3_L) using potentiometric titration. The first stage of this study was to determine the protonation constants of the ligand, which were calculated corresponding to four possible protonation steps (Table 2). The obtained values reflect the presence of two phenolic groups, one hydroxyl group, and one nitrogen donor atom capable of binding protons in aqueous solution.

**Figure 4:**
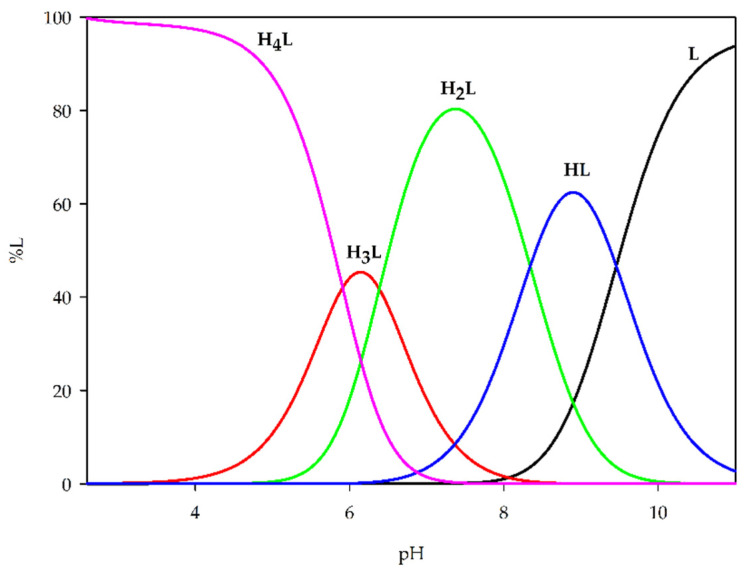
Distribution diagram of 1,3-bis(3-bromo-5-chlorosalicylideneamino)-2-propanol (H_3_L).

Analysis of these protonation constants revealed that the first deprotonation occurs at pH below 4.0 and is assigned to one of the phenolic groups, followed by subsequent protonation/deprotonation steps involving the second phenolic oxygen, hydroxyl group, and nitrogen atom. Subsequently, the obtained set of protonation constants was used to generate theoretical distribution diagrams of H_3_L species as a function of pH, providing detailed insight into the speciation profile and relative stability of the different forms of the ligand (Figure 4).

Potentiometric titrations were carried out to determine the stability of complexes formed between Eu(III), Gd(III), and Tb(III) ions and the Schiff base ligand H_3_L at metal-to-ligand ratios of 1:1 and 1:2. The overall stability constants (log*β*) as well as stepwise equilibrium constants (log*K_e_*) were calculated for the formed species (Table 3). The results show that all three lanthanide ions form stable complexes with 1,3-bis(3-bromo-5-chlorosalicylideneamino)-2-propanol, with both 1:1 and 1:2 stoichiometries being detected depending on the metal ion. The formation of M(H_x_L), M(H_x_L)_2_, ML, ML_2_, ML(OH)_x_, and ML_x_(OH)_x_ (where x = 1–3) was observed. With excess ligand concentration, the coordination shifts from simple 1:1 metal–ligand species toward complexes with two molecules of the studied ligand in the coordination sphere of the metal ion.

**Figure 5:**
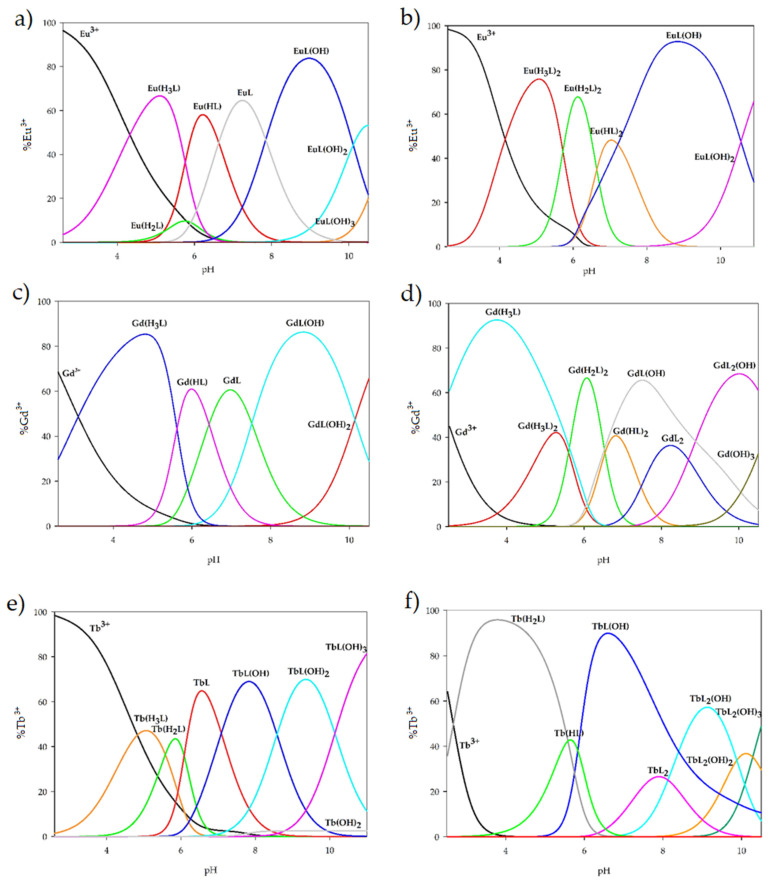
Distribution diagram of the complexes formed in the studied systems with 1,3-bis(3-bromo-5-chlorosalicylideneamino)-2-propanol and lanthanide(III) ions: (**a**) Eu(III) (1:1 ratio); (**b**) Eu(III) (1:2 ratio); (**c**) Gd(III) (1:1 ratio); (**d**) Gd(III) (1:2 ratio); (**e**) Tb(III) (1:1 ratio); (**f**) Tb(III) (1:2 ratio).

Based on the potentiometric titration data, species distribution diagrams were generated for the Ln(III)–H_3_L systems with metal-to-ligand ratios of 1:1 and 1:2, taking into account ligand protonation, metal ion hydrolysis, and the stability constants of the complexes formed (Figure 5). In the equimolar (1:1) systems, complexation starts at pH ≈ 2.5 with the formation of M(H_3_L), which reaches its maximum concentration above pH 5.0. The M(H_2_L) species was not detected for gadolinium(III); in the case of europium(III), it represents only a minor fraction (ca. 10% of the metal ions), whereas for terbium(III), it becomes the dominant form at pH 5.8, binding approximately 40% of the metal ions. With further increases in pH and progressive ligand deprotonation, M(HL) complexes appear for both gadolinium(III) and europium(III). Complete deprotonation of the ligand leads to the formation of ML species, which are dominant for all studied metal ions and at their maximum account for about 60% of all complexes present in solution. At alkaline pH, hydroxo-complexes such as ML(OH), ML(OH)_2_, and ML(OH)_3_ become predominant.

In systems with an excess of 1,3-bis(3-bromo-5-chlorosalicylideneamino)-2-propanol, differences in coordination were observed compared to systems with equal amounts of ligand. For terbium(III) ions in an acidic environment, the dominant forms are M(H_2_L) and the non-equimolar complex M(HL). At pH 6.0, the TbL_2_ form begins to form, which in alkaline pH participates in the formation of the hydroxyl complex TbL_2_(OH), whose maximum concentration is reached at pH 9.1 (about 60% of the terbium ions participate in coordination). Additionally, the formation of TbL_2_(OH)_2_ and TbL_2_(OH)_3_ hydroxocomplexes was observed. The complexation of ligands with gadolinium(III) ions at lower pH conditions is analogous to an equimolar system, where the formation of Gd(H_3_L) is observed. Unlike the equimolar system, the maximum concentration was achieved at pH 3.7, compared to pH 5.0 in the 1:1 system. With an increased pH value, the participation of two ligand molecules and the formation of Gd(H_3_L)_2_, Gd(H_2_L)_2_, Gd(HL)_2_, GdL_2_, and GdL_2_(OH) complexes were observed. The dominant forms are only Gd(H_2_L)_2_ and GdL_2_(OH) complexes, which bind approximately 65% of gadolinium(III) ions at their maximum concentration. Additionally, the GdL(OH) complex is present in the studied system, which is the dominant form in the pH range of 6.5–9.0. For europium(III) ions, the complexation process begins similarly to that in an equimolar system at a pH of approximately 2.5, but the complex formed is Eu(H_3_L)_2_. This complex dominates at a pH of 5.0, binding nearly 80% of europium(III) ions, and under the same pH conditions, the formation of the Eu(H_2_L)_2_ complex is observed. At a pH of approximately 7.0, the dominant complex is Eu(HL)_2_. A change in the conditions of the system to alkaline results in the presence of EuL(OH) hydroxy complexes (accounting for approximately 90% of all forms in the system at a pH of approximately 9.0) and EuL(OH)_2_ in the system.

The comparison of the stability of the tested complexes with our previous research [29,30,31] confirmed that the presence of Br atoms causes an increase in the equilibrium constants of formation (log*K_e_*) of the complexes, which is attributed to the effects of the heavy atom and more stable coordination in the metal environment.

### 2.3. UV-Vis Spectroscopy

**Figure 6:**
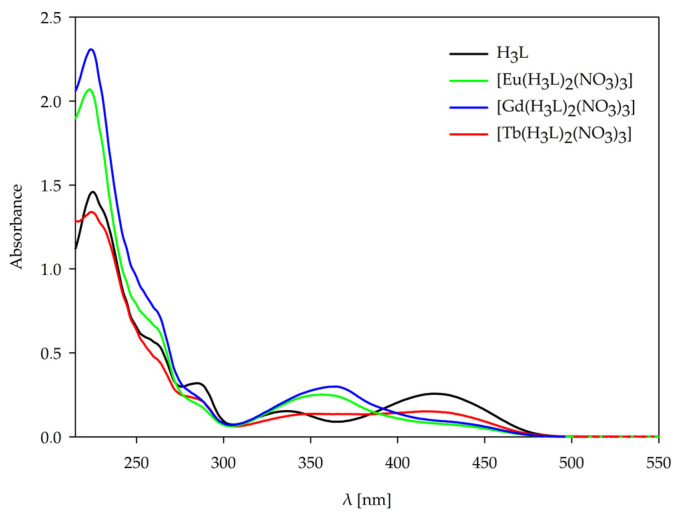
UV-Vis spectra of solid-state H_3_L ligand and its complexes with Eu(III), Gd(III), and Tb(III).

**Figure 7:**
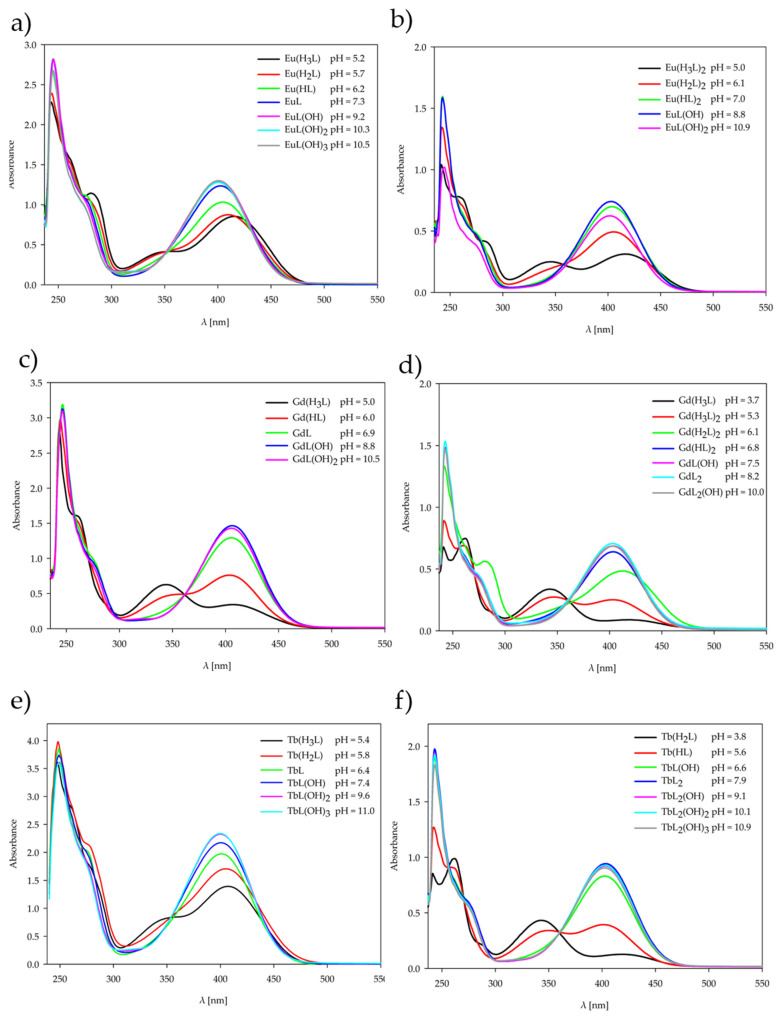
UV-Vis spectra of the complexes formed in the studied systems with 1,3-bis(3-bromo-5-chlorosalicylideneamino)-2-propanol and lanthanide(III) ions: (**a**) Eu(III) (1:1 ratio); (**b**) Eu(III) (1:2 ratio); (**c**) Gd(III) (1:1 ratio); (**d**) Gd(III) (1:2 ratio);(**e**) Tb(III) (1:1 ratio); (**f**) Tb(III) (1:2 ratio).

The behavior of the 1,3-bis(3-bromo-5-chlorosalicylideneamino)-2-propanol, H_3_L ligand, and the complexes [Eu(H_3_L)_2_(NO_3_)_3_], [Gd(H_3_L)_2_(NO_3_)_3_], [Tb(H_3_L)_2_(NO_3_)_3_] obtained in the solid state was studied using UV-Vis spectroscopy. The behavior of the H_3_L ligand and the complexes formed in the solution at various pH values was also studied using UV-Vis spectroscopy. The UV-Vis spectra were recorded in the range from 200 to 600 nm for all forms of the ligand and complexes in the solid state and at the pH value of dominance of each form determined by potentiometric titration. The coordination of the ligand H_3_L with the lanthanide ions is confirmed by the shifts in characteristic bands and changes in the absorption spectra in both solid-state and solution complexes. The observed changes for solid-state complexes and solution complexes are shown in Figure 6 and Figure 7, and all spectroscopic data are listed in Tables S3–S5. The H_3_L ligand spectrum is characterized by five main absorption bands that are assigned to the π-π* and n-π* transitions of the Schiff base H_3_L ligand. In the range of 220–300 nm, three bands occur due to the π-π* transition of the aromatic rings. The band at 337 nm is assigned to the n-π* transition of the C=N group. The last band at 423 nm may be assigned to the coupling of aromatic ring π-π* transition and an imine group n-π* transition. For the complexes, the bathochromic shifts in the last two bands are observed at 359–364 nm and 436–442 nm, which confirms the coordination of lanthanide ions with the donor nitrogen atoms of the C=N groups.

All forms of complexes formed in solution at the pH value of dominance of each form, determined by potentiometric titration, were studied by the UV-Vis spectra. For all the complexes forming in the solution, a decrease in the number of characteristic bands was observed with increasing pH values of the systems. Upon complex formation, the two higher-energy bands of the free ligand merge into a single band. The band shift, the emergence of new bands, and the variation in molar absorptivity (ε) provide evidence for the coordination of metal ions with ligands and the resulting complex formation [32]. Changes are observed above 350 nm, i.e., in the range of occurrence of characteristic bands assigned to the n-π* transition of the C=N group, for both Ln(III)–ligand 1:1 and 1:2 systems. The change in this characteristic band pattern is caused by deprotonation of the ligand with increasing pH, which correlates with a change in the internal structure of the coordination sphere. Absorption in the spectra can be ascribed to the organic ligands, as the contribution of lanthanide(III) ions is negligible owing to the Laporte-forbidden nature of their electronic transitions.

The long-term stability of the stable complexes of the tested compounds after 5 and 10 days of storage at room temperature was also examined. The changes were monitored using the UV–Vis method, and no significant differences in the spectra were observed, indicating the stability of the complexes.

### 2.4. Luminescence Spectroscopy

**Figure 8:**
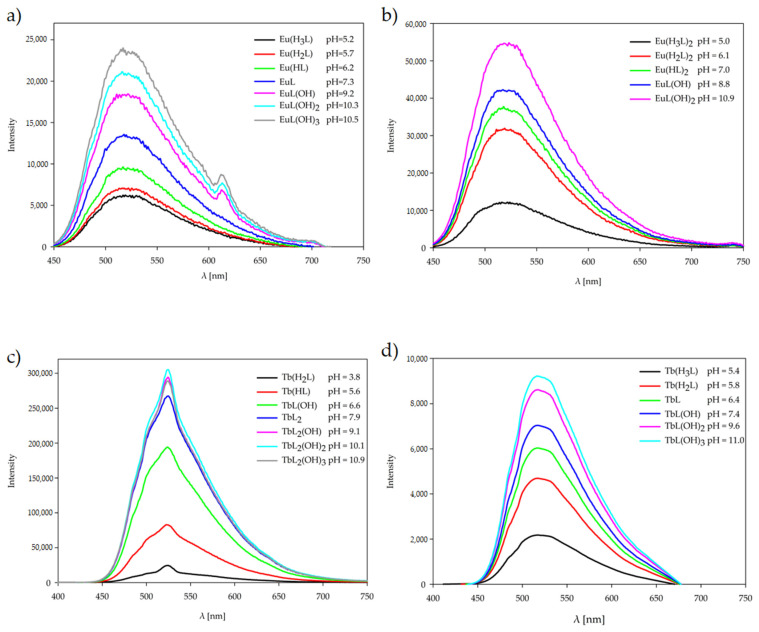
Luminescence spectra of the complexes formed in the studied systems with 1,3-bis(3-bromo-5-chlorosalicylideneamino)-2-propanol and lanthanide(III) ions: (**a**) Eu(III) (1:1 ratio); (**b**) Eu(III) (1:2 ratio); (**c**) Tb(III) (1:1 ratio); (**d**) Tb(III) (1:2 ratio).

A photoluminescence study of the ligand and its Eu(III) and Tb(III) complexes has been carried out in solution at the pH value of dominance of each form, determined by potentiometric titration, at room temperature, Figure 8. Upon excitation into the UV region, the luminescence spectrum of the H_3_L ligand in solution displays broad emission bands with maxima at 529 nm for the protonated H_4_L form of ligand (pH 4.0), 518 nm for the neutral H_3_L form (pH 6.0), 519 nm, 523 nm, and 517 nm for the deprotonated H_2_L form (pH 7.2), HL (pH 9.0), and L (pH 10.5), respectively. Luminescence studies also have been carried out, giving the emission spectra of complexes Eu(III) and Tb(III) upon excitation at 243 nm at the pH value where each form of complexes predominates. Eu(III) complexes exhibit the main emission centered around 523–516 nm, while Tb(III) complexes at around 525–515 nm, which may be caused by ligand π-π* transition. Lanthanide(III) ions present in the systems increase the luminescence of the H_3_L ligand. The enhancement value depends on the type of Ln(III) ion, but also on the deprotonation of the ligand, which depends on the pH of the tested solution. The Eu(III) complex solutions showed a very weak emission band at approximately 613 nm, attributed to the 5D^0^–7F^2^ transitions. This band is visible for complexes forming at pH above 9.2 and only for the stoichiometric ratio Ln(III):H_3_L 1:1. The results of our luminescence experiments show that low efficiency of energy transfer within the molecule occurs only in Eu(III) complexes. No transfer of energy was observed in Tb(III) complexes at any pH value. The probable cause is the large energy difference between the lowest energy level of the H_3_L ligand triplet state and the lowest resonant energy level of the Ln(III) ions studied [33].

## 3. Materials and Methods

### 3.1. Materials

Europium(III) nitrate pentahydrate, gadolinium(III) nitrate hexahydrate, and terbium(III) nitrate pentahydrate, 3-bromo-5-chloro-2-hydroxybenzaldehyde, and 2-hydroxy-1,3-propanediamine were purchased from Aldrich Chemical Company and used without further purification.

### 3.2. Synthesis

#### 3.2.1. Synthesis of 1,3-Bis(3-bromo-5-chlorosalicylideneamino)-2-propanol, H_3_L

To a 100 mL round-bottom flask were added 0.09095 g (1 mmol) of 2-hydroxy-1,3-propanediamine and 0.4706 g (2 mmol) of 3-bromo-5-chloro-2-hydroxybenzaldehyde in 40 mL of ethanol (EtOH). The reaction mixture was stirred at room temperature for 3 h. During this time, the solution gradually developed a yellow color, indicating the formation of a Schiff base. After completion of the reaction, the solvent was partially evaporated under reduced pressure to approximately half of the original volume, and the mixture was left to stand at room temperature. Upon standing, yellow single crystals suitable for X-ray diffraction analysis were formed by slow evaporation of ethanol at RT. The product, as yellow crystals, was filtered off and dried (0.2695 g, 98%). Elemental analysis for C_17_H_14_Br_2_Cl_2_N_2_O_3_ (525.02 g·mol^−1^): Found: C, 38.92; H, 2.70; N, 5.32; Calc.: C, 38.89; H, 2.69; N, 5.34%. Selected FT-IR (cm^−1^): 3254 (νOH), 3061, 2927 (νOH···N), 1637 (νC=N), 1133 (νC-O). ^1^H NMR (400 MHz, DMSO-d6) δ 14.64 (1;17) (s, 1H), 8.52 (7;11) (s, 1H), 7.71 (5;13) (dd, *J* = 2.6, 0.09 Hz, 1H), 7.5 (3;15) (d, *J* = 2.6 Hz, 1H), 5.64 (9 (O-H)) (d, *J* = 5.2 Hz, 1H), 4.06 (9) (tt, *J* = 7.6, 3.7 Hz, 1H), 3.83 (8;10) (dd, *J* = 12.9, 3.9 Hz, 1H), 3.62 (8;10) (dd, *J* = 12.8, 6.9 Hz, 1H) ^13^C NMR (101 MHz, DMSO-d6) δ 166.48 (7;11), 163.20 (1;17), 135.61 (3;15), 130.99 (5;13), 118.06 (4;14), 117.01 (6;12), 114.59 (2;16), 68.07 (9), 58.19 (8;11). ESI-MS: *m*/*z* 525 [C_17_H_13_Br_2_Cl_2_N_2_O_3_]^−^ 100%; 1084 [(C_17_H_14_Br_2_Cl_2_N_2_O_3_)_2_Cl]^−^.

#### 3.2.2. Synthesis of Europium(III) Complex [Eu(H_3_L)_2_(NO_3_)_3_]

To a 50 mL round-bottom flask equipped with a magnetic stirrer, 0.09418 g (0.4 mmol) of 3-bromo-5-chloro-2-hydroxybenzaldehyde was dissolved in 10 mL of ethanol and heated to reflux (78 °C) under continuous stirring. After 15 min, 0.0135 g (0.15 mmol) of 2-hydroxy-1,3-propanediamine, dissolved in 5 mL of ethanol, was added dropwise. The solution gradually turned yellow, indicating the formation of a Schiff base. The reaction mixture was stirred at reflux for an additional 60 min. Subsequently, 0.021403 g (0.05 mmol) of europium(III) nitrate pentahydrate, dissolved in 5 mL of ethanol, was added dropwise, and the reaction was continued under the same conditions. After 24 h, the reaction mixture was concentrated under reduced pressure to approximately 3 mL and poured into an excess of diethyl ether to induce precipitation. The resulting yellow solid was collected by decantation and dried in air at room temperature. The product was obtained as a yellow solid (0.0548 g, 80.89%). Elemental analysis for [Eu(C_17_H_14_Br_2_Cl_2_N_2_O_2_)_2_(NO_3_)_3_] (1388.02 g·mol^−1^): Found: C, 29.39; H, 2.01; N, 7.08; Calc.: C, 29.42; H, 2.02; N, 7.06%. Selected FT-IR (cm^−1^): 3064 (νOH), 1639 (νC=N), 1142 (νC-O), 1437–1206 (νNO_3_). ESI-MS: *m*/*z* 1262 [Eu(C_17_H_12_Br_2_Cl_2_N_2_O_3_)(C_17_H_13_Br_2_Cl_2_N_2_O_3_)(NO_3_)]^−^, 1199 [Eu(C_17_H_12_Br_2_Cl_2_N_2_O_3_)_2_]^−^, 736 [Eu(C_17_H_11_Br_2_Cl_2_N_2_O_3_)(NO_3_)]^−^, 586 [(C_17_H_14_Br_2_Cl_2_N_2_O_3_)(NO_3_)]^−^, 525 [C_17_H_13_Br_2_Cl_2_N_2_O_3_]^−^, 706 [Eu_2_(C_17_H_12_Br_2_Cl_2_N_2_O_3_)(C_17_H_13_Br_2_Cl_2_N_2_O_3_)(NO_3_)]^2+^, 675 [Eu(C_17_H_12_Br_2_Cl_2_N_2_O_3_)]^+^.

#### 3.2.3. Synthesis of Gadolinium(III) Complex [Gd(H_3_L)_2_(NO_3_)_3_]

To a 50 mL round-bottom flask equipped with a magnetic stirrer, 0.09484 g (0.4 mmol) of 3-bromo-5-chloro-2-hydroxybenzaldehyde was dissolved in 10 mL of absolute ethanol and heated to reflux (78 °C) under continuous stirring. After 15 min, a solution of 0.01307 g (0.15 mmol) of 2-hydroxy-1,3-propanediamine in 5 mL of ethanol was added dropwise. The reaction mixture was maintained at reflux, and the gradual development of a yellow color indicated the formation of a Schiff base. Sixty minutes after the addition of the diamine, a solution of 0.02234 g (0.05 mmol) of gadolinium(III) nitrate hexahydrate in 5 mL of ethanol was added slowly. Reflux and stirring were continued under the same conditions. After 24 h, the reaction mixture was concentrated under reduced pressure to approximately 3 mL and poured into cold diethyl ether to induce precipitation. The resulting yellow solid was collected by decantation and dried in air at room temperature. The gadolinium complex was obtained as a yellow solid (0.0353 g, 63.19%). Elemental analysis for [Gd(C_17_H_14_Br_2_Cl_2_N_2_O_2_)_2_(NO_3_)_3_] (1393.30 g·mol^−1^): Found: C, 29.29; H, 2.05; N, 7.03; Calc.: C, 29.31; H, 2.03; N, 7.04%. ESI-MS: *m*/*z* 1266 [Gd(C_17_H_12_Br_2_Cl_2_N_2_O_3_)(C_17_H_13_Br_2_Cl_2_N_2_O_3_)(NO_3_)]^−^, 1203 [Gd(C_17_H_12_Br_2_Cl_2_N_2_O_3_)_2_]^−^, 741 [Gd(C_17_H_11_Br_2_Cl_2_N_2_O_3_)(NO_3_)]^−^, 586 [(C_17_H_14_Br_2_Cl_2_N_2_O_3_)(NO_3_)]^−^, 525 [C_17_H_13_Br_2_Cl_2_N_2_O_3_]^−^, 712 [Gd_2_(C_17_H_12_Br_2_Cl_2_N_2_O_3_)(C_17_H_13_Br_2_Cl_2_N_2_O_3_)(NO_3_)]^2+^, 681 [Gd(C_17_H_12_Br_2_Cl_2_N_2_O_3_)]^+^. Selected FT-IR (cm^−1^): 3067 (νOH), 1640 (νC=N), 1146 (νC-O), 1444–1209 (νNO_3_).

#### 3.2.4. Synthesis of Terbium(III) Complex [Tb(H_3_L)_2_(NO_3_)_3_]

In a 50 mL round-bottom flask, 0.091484 g (0.4 mmol) of 3-bromo-5-chloro-2-hydroxybenzaldehyde was dissolved in 10 mL of ethanol and heated to reflux (78 °C) under continuous stirring. After 15 min, a solution of 0.0135 g (0.15 mmol) of 2-hydroxy-1,3-propanediamine in 5 mL of ethanol was added dropwise. The reaction mixture gradually turned yellow, indicating the formation of the Schiff base ligand. After an additional 60 min of reflux, 0.021751 g (0.05 mmol) of terbium(III) nitrate pentahydrate, dissolved in 5 mL of ethanol, was added slowly. After 24 h, the solution was concentrated under reduced pressure to approximately 3 mL and poured into cold diethyl ether to induce precipitation. The resulting yellow solid was collected by decantation and dried in air at room temperature. The terbium complex was obtained as a yellow solid (0.0461 g, 63.75%). [Tb(C_17_H_14_Br_2_Cl_2_N_2_O_2_)_2_(NO_3_)_3_] (1394.98 g·mol^−1^): Found: C, 29.31; H, 2.00; N, 7.05; Calc.: C, 29.27; H, 2.02; N, 7.03%. Selected FT-IR (cm^−1^): 3065 (νOH), 1640 (νC=N), 1143 (νC-O), 1440-1208 (νNO_3_). ESI-MS: *m*/*z* 1267 [Tb(C_17_H_12_Br_2_Cl_2_N_2_O_3_)(C_17_H_13_Br_2_Cl_2_N_2_O_3_)(NO_3_)]^−^, 1204 [Tb(C_17_H_12_Br_2_Cl_2_N_2_O_3_)_2_]^−^, 742 [Tb(C_17_H_11_Br_2_Cl_2_N_2_O_3_)(NO_3_)]^−^, 586 [(C_17_H_14_Br_2_Cl_2_N_2_O_3_)(NO_3_)]^−^, 525 [C_17_H_13_Br_2_Cl_2_N_2_O_3_]^−^, 713 [Tb_2_(C_17_H_12_Br_2_Cl_2_N_2_O_3_)(C_17_H_13_Br_2_Cl_2_N_2_O_3_)(NO_3_)]^2+^, 681 [Tb(C_17_H_12_Br_2_Cl_2_N_2_O_3_)]^+^.

### 3.3. Physical Measurements

Elementary analysis (CHN) was conducted using an Elementar Analyzer Vario EL III. Mass (Elementar, Langenselbold, Germany) spectrometric analyses were carried out using electrospray ionization (ESI). Measurements were performed in acetonitrile with a Waters Micromass ZQ spectrometer (Waters, Milford, MA, USA), operated in positive-ion mode. The analyte concentration was approximately 1 × 10^−4^ mol dm^−3^. Spectra were recorded in the *m*/*z* range 100–1000 with a 6 s scan time, and the final spectrum was obtained by averaging 10 scans. NMR spectra were acquired in DMSO-d_6_ on a Bruker BioSpin GmbH MHz spectrometer (Bruker BioSpin GmbH, Ettlingen, Germany), calibrated against residual solvent signals (δ = 2.50 ppm). Infrared spectra were collected with an INVENIO R Bruker FT-IR spectrometer (Bruker, Ettlingen, Germany), and band positions are reported in cm^−1^. Electronic absorption spectra were measured on a Nicolet Evolution 300 UV–VIS ThermoFisher Scientific spectrophotometer (ThermoFisher Scientific, Waltham, MA, USA) in ethanol and a mixture of dimethyl sulfoxide and water. The luminescence studies were recorded on an RF−6000 spectrofluorophotometer (Shimadzu, Kyoto, Japan) using 5/5 nm slit widths.

### 3.4. Equilibrium Studies

All experiments were conducted in a dimethyl sulfoxide/water mixture (20:80, *v*/*v*) prepared with demineralized, CO_2_-free water. Potentiometric titrations were performed on a Metrohm Titrino 702 (Metrohm, Herisau, Switzerland) with an autoburette and a Metrohm Solvotrode combination pH electrode designed for nonaqueous titrations, calibrated prior to each run. pH readings were corrected before every measurement series using two standard buffers (pH 4.002 and 9.225). Titrations were carried out under helium (5.0) at constant ionic strength (0.1 M LiNO_3_) and temperature (20.0 °C), with CO_2_-free NaOH (0.1979 M) as titrant. The ligand concentration was 1 × 10^−3^ M, and HCl (1.5 mL, 0.1 M) was added to the systems. Metal-to-ligand ratios of 1:1 and 1:2 were investigated. The pK_w_ value determined for the DMSO/water (20:80) mixture was 14.501 [34]. Protonation constants of 1,3-bis(3-bromo-5-chlorosalicylideneamino)-2-propanol, model selection, and complex stability constants were obtained using HYPERQUAD software (version 5.2.0.19), which applies a nonlinear least-squares approach [35]. Species distribution diagrams were generated with the HySS (Hyperquad Simulation and Speciation) program (version 4.0.31) [36]. Calculations were based on 150–350 experimental points, excluding sections of titrations where precipitation occurred. Model construction began with the simplest assumptions and was progressively expanded; species rejected during refinement were eliminated. The verification criteria followed those outlined in previous studies [37,38].

### 3.5. Crystal Structure

Single-crystal X-ray diffraction data were collected at 100(1) K on a Bruker D8 QUEST KAPPA diffractometer, equipped with a microfocus sealed tube (CuK_α_ radiation, λ = 1.54178 Å), using a multilayer mirror as monochromator and a Bruker PHOTON III CPAD detector (Bruker, Ettlingen, Germany). The data collection temperature was controlled by an Oxford Cryostream 700 low temperature device (Oxford Cryosystems Ltd., Oxford, UK). All data were integrated with SAINT V8.41 [39]. A multi-scan absorption correction using SADABS 2016/2 was applied [40]. The primary computational tool used was OLEX2 (version 1.5) [41]. The structures were solved with ShelxT (version 2014/7) [42] and refined through full-matrix least-squares method on F^2^ using SHELXL-2015/2017, employing scattering factors from SHELXL [43]. All non-hydrogen atoms were refined with anisotropic displacement parameters. All hydrogen atoms were refined with isotropic displacement parameters. Some of their coordinates were refined freely, and some were calculated positions using a riding model, with their Uiso values constrained to 1.2 times their carrier atoms. One atom has been refined as disordered between O35 and N32 (cf. Discussion). Structure representation was prepared with Olec1.5 [41] and Mercury 4.3.0 software [44]. Table 4 lists the relevant experimental data and refinement details. Crystallographic data for the structural analysis have been deposited with the Cambridge Crystallographic Data Centre, No CCDC 2435763. Copies of this information may be obtained free of charge from: The Director, CCDC, 12 Union Road, Cambridge, CB2 1EZ, UK. e-mail: deposit@ccdc.cam.ac.uk or www: http://www.ccdc.cam.ac.uk.

## 4. Conclusions

The Schiff base ligand H_3_L, 1,3-bis(3-bromo-5-chlorosalicylideneamino)-2-propanol and its complexes of lanthanide(III) ions with formulas [Eu(H_3_L)_2_(NO_3_)_3_], [Gd(H_3_L)_2_(NO_3_)_3_], [Tb(H_3_L)_2_(NO_3_)_3_], where were synthesized. The solid-state complexes were obtained by template reaction of 3-bromo-5-chloro-2-hydroxybenzaldehyde with 2-hydroxy-1,3-propanediamine in the presence of an appropriate lanthanide(III) ion. It was found that the crystal structure of 1,3-bis(3-bromo-5-chlorosalicylideneamino)-2-propanol is very similar to that of the one already published 1,3-bis(3,5-dibromosalicylideneamino)-2-propanol. In both cases, the asymmetric parts of the appropriate unit cells consist of three independent molecules, which occupy analogous positions. Potentiometric titrations were carried out to determine the stability of complexes formed between Eu(III), Gd(III), and Tb(III) ions and the Schiff base ligand H_3_L at metal-to-ligand ratios of 1:1 and 1:2. The overall stability constants (log*β*) as well as stepwise equilibrium constants (log*K_e_*) were calculated for the formed species. The results show that all three lanthanide(III) ions form stable complexes with 1,3-bis(3-bromo-5-chlorosalicylideneamino)-2-propanol, with both 1:1 and 1:2 stoichiometries being detected depending on the metal ion. Spectroscopic studies confirmed the coordination of the lanthanide(III) ion by the donor nitrogen and oxygen atoms of the H_3_L ligand and the complementing of the coordination sphere by the bidentate coordination of nitrate groups. Luminescence studies showed that low efficiency of energy transfer within the molecule occurs only in Eu(III) complexes. No energy transfer was observed in Tb(III) complexes at any pH value. The probable cause is the large energy difference between the lowest energy level of the H_3_L ligand triplet state and the lowest resonant energy level of the Ln(III) ions studied. Organic ligands can act as energy acceptors from lanthanide(III) ions, leading to a weakening or complete quenching of luminescence.

## Figures and Tables

**Table 1:** The geometry of selected hydrogen bonds.

*D*—H···*A*	*D*—H (Å)	H···*A* (Å)	*D*···*A* (Å)	*D*—H···*A* (°)
O10—H10···O21	0.84	2.01	2.836 (5)	166.7
N12—H12···O15	0.88	1.88	2.599 (5)	137.1
N48—H48···O41	0.88	1.94	2.619 (6)	133.1
N28—H28···O21	0.88	1.96	2.630 (5)	132.4
N28—H28···O54	0.88	2.27	2.917 (5)	130.7
N8—H8···O1	0.88	1.87	2.569 (5)	134.9
O30—H30···O1	0.84	1.96	2.774 (5)	162.0
N32—H32···O35	0.88	1.82	2.532 (5)	137.1
N52—H52···O21	0.88	2.59	3.222 (6)	129.2
N52—H52···O54	0.88	1.83	2.557 (5)	138.1
O50—H50···O15	0.84	1.91	2.754 (5)	178.3

**Table 2:** Overall protonation constants (log*β*) and successive protonation constants (log*K_e_*) of 1,3-bis(3-bromo-5-chlorosalicylideneamino)-2-propanol (H_3_L).

Species	log*β*	log*K_e_*
H_4_L	30.09 (3)	5.91
H_3_L	24.19 (3)	6.39
H_2_L	17.79 (2)	8.34
HL	9.46 (2)	9.46

**Table 3:** The overall stability constants (log*β*) and the equilibrium constants of formation (log*K_e_*) of the complexes formed in the studied systems with 1,3-bis(3-bromo-5-chlorosalicylideneamino)-2-propanol and lanthanide ions (standard deviations are given in parentheses).

Species		Eu(III)		Gd(III)		Tb(III)	
		log*β*	log*K_e_*	log*β*	log*K_e_*	log*β*	log*K_e_*
**1:1**	M(H_3_L)	28.65 (4)	4.86	29.88 (5)	6.09	28.26 (7)	4.47
	M(H_2_L)	22.31 (1)	4.78	-	-	22.68 (9)	5.15
	M(HL)	17.13 (4)	7.77	18.64 (5)	9.28	-	-
	ML	10.50 (6)	10.50	12.20 (5)	12.20	10.51 (1)	10.51
	ML(OH)	2.65 (4)	6.66	4.68 (5)	6.98	3.42 (4)	7.41
	ML(OH)_2_	−7.46 (5)	4.39	−5.46 (6)	4.36	−5.16 (8)	5.92
	ML(OH)_3_	−18.39 (7)	3.57	-	-	−15.30 (9)	4.36
**1:2**	M(H_3_L)_2_	57.71 (7)	10.13	58.47 (1)	10.90	-	-
	M(H_2_L)_2_	46.27 (7)	11.20	47.28 (8)	12.22	-	-
	M(HL)_2_	32.99 (8)	14.27	34.19 (1)	15.48	-	-
	ML_2_	-	-	19.25 (1)	19.25	23.36 (8)	23.36
	M(H_3_L)	-	-	-	-	30.43 (8)	6.65
	M(H_2_L)	-	-	-	-	27.47 (4)	9.94
	M(HL)	-	-	-	-	21.89 (5)	12.53
	ML_2_(OH)	-	-	10.55 (8)	5.80	15.23 (6)	6.37
	ML_2_(OH)_2_	-	-	-	-	5.35 (7)	4.62
	ML_2_(OH)_3_	-	-	-	-	−4.93 (7)	4.22
	ML(OH)	4.46 (8)	18.96	6.13 (1)	20.63	10.23 (5)	24.73
	ML(OH)_2_	−6.09 (1)	3.95	-	-	-	-

Hydrolysis constants used in calulations: Eu(OH)_2_ = −15.15; Eu(OH)_3_ = −24.31; Gd(OH) = −9.30; Gd(OH)_2_ = −17.74; Gd(OH)_3_ = −28.12; Tb(OH)_2_ = −15.56.

**Table 4:** Experimental details.

	1,3-Bis(3-bromo-5-chlorosalicylideneamino)-2-propanol
Crystal data	
Chemical formula	3(C_17_H_14_Br_2_Cl_2_N_2_O_3_)
*M*_r_	1575.06
Crystal system, space group	Monoclinic, *P*2_1_/*n*
Temperature (K)	100
*a*, *b*, *c* (Å)	16.445 (3), 18.111 (3), 18.956 (4)
â (°)	92.978 (9)
*V* (Å^3^)	5638.2 (17)
*Z*	4
Radiation type	Cu *K*
m (mm^−1^)	8.27
Crystal size (mm)	0.2 × 0.05 × 0.05
Data collection	
Diffractometer	Bruker D8 QUEST
Absorption correction	Multi-scan
*T*_min_, *T*_max_	0.250, 0.383
No. of measured, independent and observed [*I* > 2ó(*I*)] reflections	42,240, 10,291, 8425
*R*_int_	0.064
(sinθ/λ_max_ (Å^−1^)	0.603
Refinement	
*R*[*F*^2^ > 2ó(*F*^2^)], *wR*(*F*^2^), *S*	0.046, 0.113, 1.02
No. of reflections	10,291
No. of parameters	802
Δρ_max_, Δρ_min_ (e Å^−3^)	1.56, −1.17

## Data Availability

The original contributions presented in this study are included in the article/Supplementary Materials. Further inquiries can be directed to the corresponding author.

## Supplementary Materials

The following supporting information can be downloaded at: https://www.mdpi.com/article/10.3390/ijms262110379/s1.
