# Supplementary material for: Potentiometric Studies of the Complexation Properties of Selected Lanthanide Ions with Schiff Base Ligand
Source: Int J Mol Sci. 2025 Oct 25;26(21):10379. doi: 10.3390/ijms262110379 (PMC12609344; doi:10.3390/ijms262110379)
Supplement: Supplementary file 1 [file ijms-26-10379-s001.zip › ijms-3934014-supplementary.pdf]

Potentiometric studies of the complexation properties of selected lanthanide ions with Schiff base ligand

Julia Barańska, Katarzyna Koroniak-Szejn, Michał Zabiszak, Anita Grześkiewicz Monika Skrobańska, Martyna Nowak, Renata Jastrzab, Małgorzata T. Kaczmarek*

Suplementarny materials

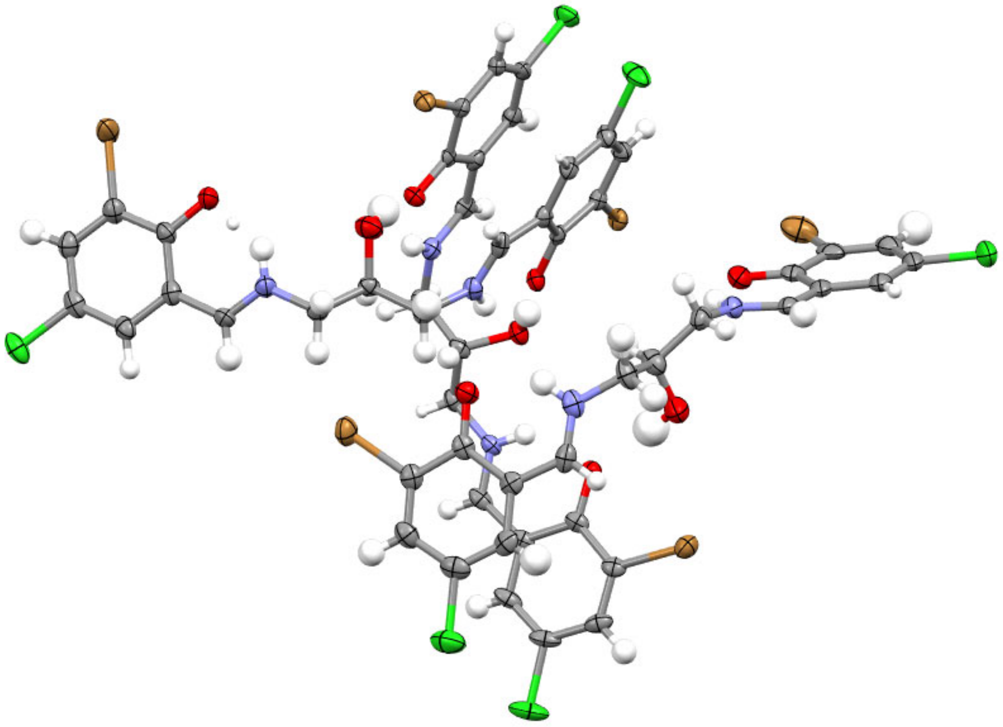

Figure S1. Perspective view of the asymmetric part of unit cell

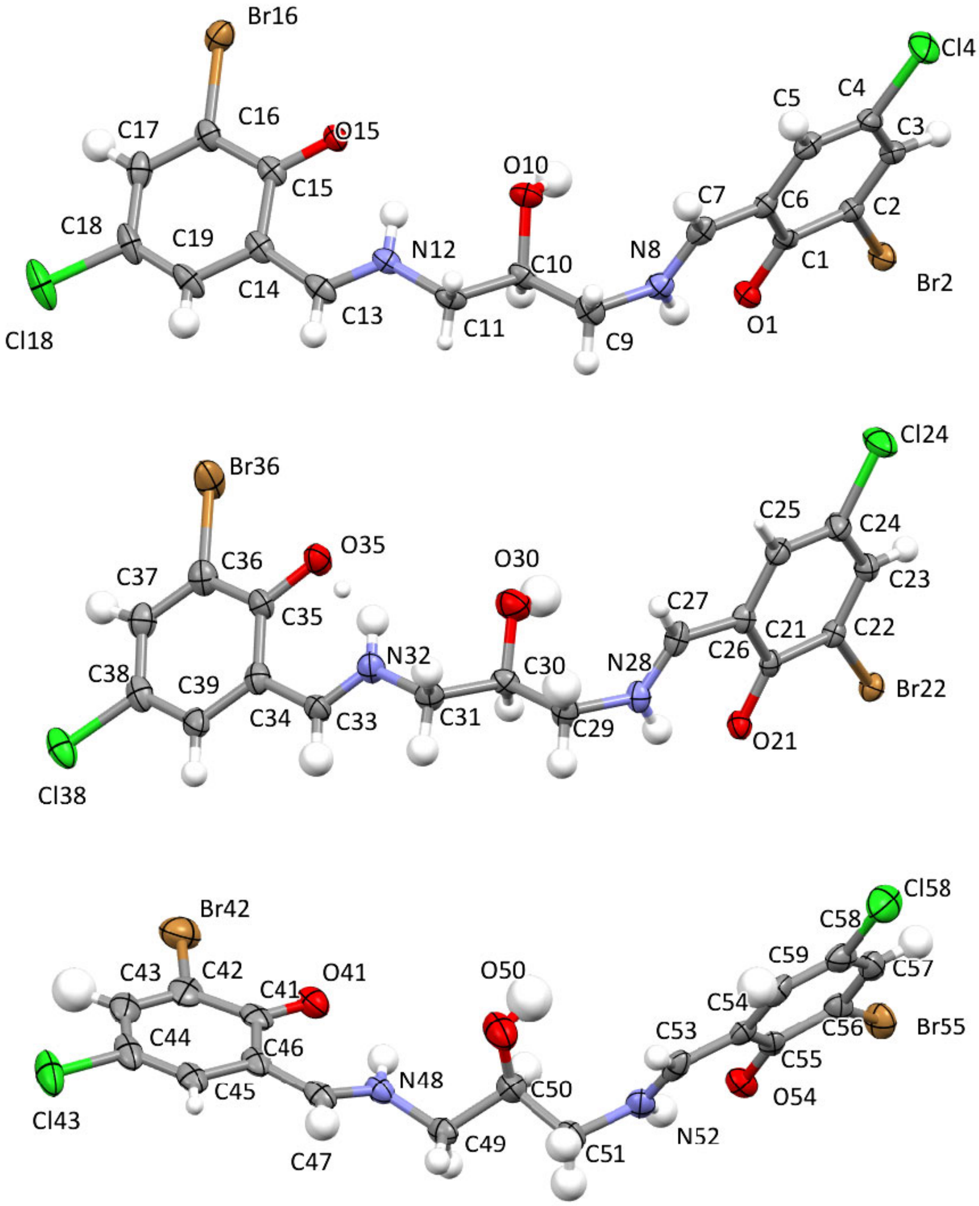

Figure S2. Perspective view of the *N*,*N′*-bis(3-bromo-5-chlorosalicylidene)-2-hydroxy-1,3-propanediamine molecules with numbering scheme, the ellipsoids are drawn at the 50% probability level, and hydrogen atoms are shown as spheres of arbitrary radii. The following colours have been assigned to the atoms: light grey-carbon, white-hydrogen, blue-nitrogen, red-oxygen, green-chlorine, brown -bromine

Table S1. Selected geometric parameters (Å, º)

Br22—C22	1.891 (4)	C6—C5	1.412 (7)
Br2—C2	1.885 (4)	C6—C7	1.410 (6)
Br16—C16	1.895 (5)	C15—C14	1.441 (7)

Br55—C56	1.906 (5)	C15—C16	1.436 (7)
Br36—C36	1.890 (5)	C26—C27	1.426 (6)
Br42—C42	1.892 (6)	C13—C14	1.426 (7)
Cl38—C38	1.742 (5)	C22—C23	1.375 (7)
Cl4—C4	1.743 (5)	C55—C54	1.444 (7)
Cl24—C24	1.748 (5)	C55—C56	1.425 (7)
Cl18—C18	1.740 (5)	C23—C24	1.408 (7)
Cl43—C44	1.751 (6)	C9—C10	1.520 (7)
Cl58—C58	1.743 (6)	C19—C14	1.404 (7)
O1—C1	1.291 (5)	C19—C18	1.355 (8)
O15—C15	1.274 (5)	C11—C10	1.521 (6)
O21—C21	1.281 (5)	C5—C4	1.369 (7)
O10—C10	1.435 (5)	C16—C17	1.369 (7)
O54—C55	1.273 (6)	C30—C29	1.532 (7)
O35—C35	1.336 (6)	C30—C31	1.522 (6)
O41—C41	1.274 (6)	C47—C46	1.410 (8)
N12—C13	1.290 (6)	C59—C54	1.398 (7)
N12—C11	1.455 (6)	C59—C58	1.352 (8)
N48—C47	1.293 (6)	C33—C34	1.448 (7)
N48—C49	1.452 (7)	C35—C34	1.408 (7)
N28—C27	1.282 (6)	C35—C36	1.401 (7)
N28—C29	1.453 (6)	C34—C39	1.410 (7)
N8—C9	1.455 (6)	C53—C54	1.427 (7)
N8—C7	1.293 (6)	C17—C18	1.403 (8)
O30—C30	1.396 (6)	C57—C56	1.363 (8)
N32—C33	1.270 (6)	C57—C58	1.404 (8)
N32—C31	1.440 (6)	C46—C41	1.438 (8)
C1—C2	1.420 (6)	C46—C45	1.418 (7)
C1—C6	1.438 (6)	C39—C38	1.367 (7)
N52—C53	1.285 (7)	C49—C50	1.517 (7)
N52—C51	1.453 (7)	C38—C37	1.391 (8)
C3—C2	1.367 (6)	C36—C37	1.378 (8)
C3—C4	1.395 (7)	C41—C42	1.442 (8)
O50—C50	1.357 (7)	C51—C50	1.527 (8)
C25—C26	1.408 (7)	C45—C44	1.351 (8)
C25—C24	1.368 (7)	C43—C42	1.366 (9)

C21—C26	1.441 (6)	C43—C44	1.398 (9)
C21—C22	1.435 (6)		
C13—N12—C11	126.6 (4)	C25—C24—Cl24	120.1 (4)
C47—N48—C49	122.7 (5)	C25—C24—C23	121.4 (5)
C27—N28—C29	124.0 (4)	C23—C24—Cl24	118.5 (4)
C7—N8—C9	124.4 (4)	C58—C59—C54	120.8 (5)
C33—N32—C31	121.5 (4)	N32—C33—C34	121.0 (5)
O1—C1—C2	122.6 (4)	O35—C35—C34	121.7 (4)
O1—C1—C6	121.9 (4)	O35—C35—C36	120.3 (4)
C2—C1—C6	115.4 (4)	C36—C35—C34	117.9 (4)
C53—N52—C51	126.4 (5)	C35—C34—C33	119.8 (4)
C2—C3—C4	119.4 (4)	C35—C34—C39	120.1 (5)
C1—C2—Br2	116.6 (3)	C39—C34—C33	120.1 (5)
C3—C2—Br2	120.2 (3)	N52—C53—C54	122.3 (5)
C3—C2—C1	123.1 (4)	C16—C17—C18	119.9 (5)
C24—C25—C26	119.5 (4)	C59—C54—C55	121.5 (5)
O21—C21—C26	122.1 (4)	C59—C54—C53	119.8 (5)
O21—C21—C22	123.3 (4)	C53—C54—C55	118.7 (5)
C22—C21—C26	114.7 (4)	C56—C57—C58	119.1 (5)
C5—C6—C1	121.2 (4)	C47—C46—C41	120.4 (5)
C7—C6—C1	119.6 (4)	C47—C46—C45	117.7 (5)
C7—C6—C5	119.2 (4)	C45—C46—C41	121.7 (5)
O15—C15—C14	122.4 (4)	N28—C29—C30	109.8 (4)
O15—C15—C16	122.4 (4)	C38—C39—C34	119.6 (5)
C16—C15—C14	115.2 (4)	N48—C49—C50	113.5 (4)
C25—C26—C21	122.1 (4)	C39—C38—Cl38	119.0 (4)
C25—C26—C27	117.9 (4)	C39—C38—C37	121.6 (5)
C27—C26—C21	119.9 (4)	C37—C38—Cl38	119.4 (4)
N12—C13—C14	122.6 (5)	C35—C36—Br36	118.0 (4)
C21—C22—Br22	118.0 (3)	C37—C36—Br36	120.1 (4)
C23—C22—Br22	118.8 (3)	C37—C36—C35	121.9 (5)
C23—C22—C21	123.2 (4)	C55—C56—Br55	116.0 (4)
N28—C27—C26	124.5 (5)	C57—C56—Br55	119.9 (4)
O54—C55—C54	122.9 (4)	C57—C56—C55	124.1 (5)
O54—C55—C56	123.1 (4)	N32—C31—C30	111.2 (4)

C56—C55—C54	114.0 (4)	C36—C37—C38	118.8 (5)
C22—C23—C24	119.2 (4)	O41—C41—C46	122.6 (5)
N8—C9—C10	110.4 (4)	O41—C41—C42	123.6 (5)
C18—C19—C14	121.4 (5)	C46—C41—C42	113.8 (5)
N12—C11—C10	109.8 (4)	N52—C51—C50	110.6 (4)
C4—C5—C6	119.4 (4)	C44—C45—C46	120.1 (6)
C13—C14—C15	120.1 (4)	C59—C58—Cl58	121.3 (5)
C19—C14—C15	120.6 (5)	C59—C58—C57	120.6 (5)
C19—C14—C13	119.3 (5)	C57—C58—Cl58	118.1 (4)
N8—C7—C6	123.3 (4)	C19—C18—Cl18	121.7 (4)
C15—C16—Br16	117.9 (3)	C19—C18—C17	120.3 (5)
C17—C16—Br16	119.4 (4)	C17—C18—Cl18	118.0 (4)
C17—C16—C15	122.7 (5)	C42—C43—C44	119.4 (6)
O30—C30—C29	109.6 (4)	O50—C50—C49	109.0 (4)
O30—C30—C31	107.3 (4)	O50—C50—C51	111.5 (5)
C31—C30—C29	108.8 (4)	C49—C50—C51	107.8 (4)
N48—C47—C46	123.3 (5)	C41—C42—Br42	117.1 (4)
C3—C4—Cl4	118.8 (4)	C43—C42—Br42	119.1 (5)
C5—C4—Cl4	119.7 (4)	C43—C42—C41	123.8 (6)
C5—C4—C3	121.4 (4)	C45—C44—Cl43	119.7 (5)
O10—C10—C9	110.0 (4)	C45—C44—C43	121.2 (5)
O10—C10—C11	105.9 (4)	C43—C44—Cl43	119.1 (5)
C9—C10—C11	109.6 (4)		
Br22—C22—C23—C24	179.3 (4)	C22—C23—C24—C25	-0.4 (7)
Br16—C16—C17—C18	-179.8 (4)	C27—N28—C29—C30	93.0 (6)
Br36—C36—C37—C38	179.0 (4)	C9—N8—C7—C6	-175.0 (4)
Cl38—C38—C37—C36	177.7 (4)	C11—N12—C13—C14	177.1 (4)
O1—C1—C2—Br2	-1.3 (5)	C5—C6—C7—N8	-178.5 (4)
O1—C1—C2—C3	-179.4 (4)	C14—C15—C16—Br16	-178.6 (3)
O1—C1—C6—C5	179.2 (4)	C14—C15—C16—C17	0.5 (6)
O1—C1—C6—C7	-0.7 (6)	C14—C19—C18—Cl18	-178.7 (4)
O15—C15—C14—C13	-2.0 (7)	C14—C19—C18—C17	0.7 (8)
O15—C15—C14—C19	178.9 (4)	C7—N8—C9—C10	97.8 (5)
O15—C15—C16—Br16	1.0 (6)	C7—C6—C5—C4	-179.6 (4)
O15—C15—C16—C17	180.0 (4)	C16—C15—C14—C13	177.5 (4)

O21—C21—C26—C25	179.0 (4)	C16—C15—C14—C19	-1.5 (6)
O21—C21—C26—C27	-6.4 (6)	C16—C17—C18—Cl18	177.6 (4)
O21—C21—C22—Br22	1.6 (6)	C16—C17—C18—C19	-1.8 (7)
O21—C21—C22—C23	-180.0 (4)	C47—N48—C49—C50	-133.3 (5)
O54—C55—C54—C59	178.4 (4)	C47—C46—C41—O41	-4.1 (7)
O54—C55—C54—C53	-1.3 (7)	C47—C46—C41—C42	174.9 (4)
O54—C55—C56—Br55	-0.4 (6)	C47—C46—C45—C44	-174.6 (5)
O54—C55—C56—C57	-179.6 (5)	C4—C3—C2—Br2	-178.0 (3)
O35—C35—C34—C33	-1.7 (7)	C4—C3—C2—C1	0.0 (7)
O35—C35—C34—C39	178.1 (4)	C24—C25—C26—C21	1.1 (7)
O35—C35—C36—Br36	2.9 (6)	C24—C25—C26—C27	-173.6 (4)
O35—C35—C36—C37	-177.2 (5)	C33—N32—C31—C30	126.6 (5)
O41—C41—C42—Br42	-0.4 (7)	C33—C34—C39—C38	179.0 (4)
O41—C41—C42—C43	178.9 (5)	C35—C34—C39—C38	-0.9 (7)
N12—C13—C14—C15	2.1 (7)	C35—C36—C37—C38	-1.0 (8)
N12—C13—C14—C19	-178.9 (4)	C34—C35—C36—Br36	-177.6 (4)
N12—C11—C10—O10	57.1 (5)	C34—C35—C36—C37	2.3 (7)
N12—C11—C10—C9	175.7 (4)	C34—C39—C38—Cl38	-176.8 (4)
N48—C47—C46—C41	-2.1 (7)	C34—C39—C38—C37	2.3 (7)
N48—C47—C46—C45	173.3 (5)	C53—N52—C51—C50	94.4 (6)
N48—C49—C50—O50	53.0 (6)	C54—C55—C56—Br55	179.2 (3)
N48—C49—C50—C51	174.1 (5)	C54—C55—C56—C57	0.0 (7)
N8—C9—C10—O10	-60.6 (5)	C54—C59—C58—Cl58	178.3 (4)
N8—C9—C10—C11	-176.7 (4)	C54—C59—C58—C57	-0.6 (8)
O30—C30—C29—N28	-61.5 (5)	C46—C41—C42—Br42	-179.4 (3)
O30—C30—C31—N32	59.8 (5)	C46—C41—C42—C43	-0.1 (7)
N32—C33—C34—C35	4.2 (7)	C46—C45—C44—Cl43	179.2 (4)
N32—C33—C34—C39	-175.6 (5)	C46—C45—C44—C43	-0.7 (8)
C1—C6—C5—C4	0.4 (7)	C29—N28—C27—C26	-171.0 (4)
C1—C6—C7—N8	1.4 (7)	C29—C30—C31—N32	178.4 (4)
N52—C53—C54—C55	-1.1 (7)	C39—C38—C37—C36	-1.4 (7)
N52—C53—C54—C59	179.2 (5)	C49—N48—C47—C46	-172.1 (5)
N52—C51—C50—O50	-75.0 (6)	C36—C35—C34—C33	178.8 (4)
N52—C51—C50—C49	165.5 (5)	C36—C35—C34—C39	-1.4 (7)
C2—C1—C6—C5	0.3 (6)	C56—C55—C54—C59	-1.3 (6)
C2—C1—C6—C7	-179.6 (4)	C56—C55—C54—C53	179.0 (4)

C2—C3—C4—Cl4	179.0 (3)	C56—C57—C58—Cl58	-179.5 (4)
C2—C3—C4—C5	0.8 (7)	C56—C57—C58—C59	-0.6 (8)
C25—C26—C27—N28	177.4 (4)	C31—N32—C33—C34	177.8 (4)
C21—C26—C27—N28	2.5 (7)	C31—C30—C29—N28	-178.6 (4)
C21—C22—C23—C24	0.9 (7)	C41—C46—C45—C44	0.8 (7)
C6—C1—C2—Br2	177.5 (3)	C51—N52—C53—C54	-176.5 (4)
C6—C1—C2—C3	-0.6 (6)	C45—C46—C41—O41	-179.4 (5)
C6—C5—C4—Cl4	-179.2 (3)	C45—C46—C41—C42	-0.4 (7)
C6—C5—C4—C3	-1.0 (7)	C58—C59—C54—C55	1.6 (7)
C15—C16—C17—C18	1.2 (7)	C58—C59—C54—C53	-178.7 (5)
C26—C25—C24—Cl24	178.2 (4)	C58—C57—C56—Br55	-178.3 (4)
C26—C25—C24—C23	-0.6 (7)	C58—C57—C56—C55	0.8 (8)
C26—C21—C22—Br22	-178.8 (3)	C18—C19—C14—C15	1.0 (7)
C26—C21—C22—C23	-0.3 (6)	C18—C19—C14—C13	-178.0 (5)
C13—N12—C11—C10	143.4 (5)	C42—C43—C44—Cl43	-179.7 (4)
C22—C21—C26—C25	-0.7 (6)	C42—C43—C44—C45	0.2 (8)
C22—C21—C26—C27	174.0 (4)	C44—C43—C42—Br42	179.5 (4)
C22—C23—C24—Cl24	-179.2 (4)	C44—C43—C42—C41	0.2 (8)

Table S2. Selected hydrogen-bond parameters

D—H···*A*	*D*—H (Å)	H···*A* (Å)	*D*···*A* (Å)	*D*—H···*A* (°)
O10—H10···Br22	0.84	3.07	3.587 (3)	121.9
O10—H10···O21	0.84	2.01	2.836 (5)	166.7
N12—H12···O15	0.88	1.88	2.599 (5)	137.1
N48—H48···Br22	0.88	2.99	3.504 (4)	119.0
N48—H48···O41	0.88	1.94	2.619 (6)	133.1
N28—H28···O21	0.88	1.96	2.630 (5)	132.4
N28—H28···O54	0.88	2.27	2.917 (5)	130.7
N8—H8···O1	0.88	1.87	2.569 (5)	134.9
O30—H30···Br2	0.84	2.77	3.278 (4)	120.6
O30—H30···O1	0.84	1.96	2.774 (5)	162.0
N32—H32···O35	0.88	1.82	2.532 (5)	137.1
N52—H52···O21	0.88	2.59	3.222 (6)	129.2
N52—H52···O54	0.88	1.83	2.557 (5)	138.1

O50—H50···O15	0.84	1.91	2.754 (5)	178.3
C10—H10A···O54	1.00	2.31	3.225 (6)	152.5
C57—H57···Br42^{i}	0.95	2.83	3.609 (6)	139.8
C31—H31B···Cl4^{ii}	0.99	2.75	3.357 (5)	119.9
C49—H49A···Br36^{iii}	1.05 (5)	3.03 (5)	3.684 (5)	121 (3)
C49—H49A···O35^{iii}	1.05 (5)	2.17 (5)	3.203 (6)	168 (4)
C29—H29A···O54	0.94 (6)	2.49 (6)	3.109 (7)	124 (4)
C53—H53···Br42^{ii}	0.91 (5)	3.11 (5)	3.731 (5)	127 (4)
C49—H49B···Br22	0.92 (6)	3.11 (5)	3.628 (5)	118 (4)
C29—H29B···Cl18^{iv}	1.01 (7)	2.72 (7)	3.655 (6)	153 (5)
C11—H11B···O15^{i}	0.97 (5)	2.59 (5)	3.481 (6)	152 (4)
C13—H13···O41^{i}	0.87 (5)	2.59 (6)	3.438 (6)	164 (4)
C9—H9B···Br2^{v}	0.95 (5)	3.05 (5)	3.616 (5)	120 (4)
C19—H19···Cl38^{vi}	0.80 (6)	2.98 (6)	3.740 (6)	162 (5)
O35—H35···N32	0.88 (10)	1.69 (9)	2.532 (5)	160 (9)

Symmetry code(s): (i) -*x*+1, -*y*+1, -*z*+1; (ii) *x*+1/2, -*y*+1/2, *z*-1/2; (iii) *x*+1/2, -*y*+1/2, *z*+1/2; (iv) -*x*+3/2, *y*-1/2, -*z*+1/2; (v) -*x*+1/2, *y*+1/2, -*z*+1/2; (vi) *x*+1/2, -*y*+3/2, *z*+1/2.

Table S3. Absorption data for the complexes formed in the studied systems (1:1 ratio)

Species			pH	A	λ_{max} (nm)	ε ($dm^3 \cdot mol^{-1} \cdot cm^{-1}$)
1:1	Eu(III)	$M(H_3L)$	5.2	2.30	243	$4.60 \cdot 10^7$
				1.16	280	$2.32 \cdot 10^7$
				0.43	350	$8.60 \cdot 10^6$
				0.86	414	$1.72 \cdot 10^7$
		$M(H_2L)$	5.7	2.40	244	$4.80 \cdot 10^7$
				1.07	279	$2.14 \cdot 10^7$
				0.44	354	$8.80 \cdot 10^6$
				0.90	410	$1.80 \cdot 10^7$
		M(HL)	6.2	2.65	243	$5.30 \cdot 10^7$
				1.12	277	$2.24 \cdot 10^7$
				1.04	405	$2.08 \cdot 10^7$
		ML	7.3	2.83	245	$5.66 \cdot 10^7$
				1.08	276	$2.16 \cdot 10^7$
				1.24	403	$2.48 \cdot 10^7$
		ML(OH)	9.2	2.81	245	$5.62 \cdot 10^7$
				1.13	272	$2.26 \cdot 10^7$
				1.27	402	$2.54 \cdot 10^7$
		$ML(OH)_2$	10.3	2.66	245	$5.32 \cdot 10^7$
				1.32	400	$2.64 \cdot 10^7$
		$ML(OH)_3$	10.5	2.67	245	$5.34 \cdot 10^7$
				1.31	400	$2.62 \cdot 10^7$

	Gd(III)	$M(H_3L)$	5.0	2.83	241	$5.66 \cdot 10^7$
				1.60	262	$3.20 \cdot 10^7$
				0.64	344	$1.28 \cdot 10^7$
				0.36	412	$7.20 \cdot 10^6$
		M(HL)	6.0	2.98	244	$5.96 \cdot 10^7$
				1.51	260	$3.02 \cdot 10^7$
				0.48	344	$9.60 \cdot 10^6$
				0.77	404	$1.54 \cdot 10^7$
		ML	6.9	3.19	247	$6.38 \cdot 10^7$
				1.00	275	$2.00 \cdot 10^7$
				1.28	407	$2.56 \cdot 10^7$
		ML(OH)	8.8	3.13	246	$6.26 \cdot 10^7$
				0.98	273	$1.96 \cdot 10^7$
				1.47	406	$2.94 \cdot 10^7$
		$ML(OH)_2$	10.5	3.09	244	$6.18 \cdot 10^7$
				0.92	276	$1.84 \cdot 10^7$
				1.42	407	$2.84 \cdot 10^7$
	Tb(III)	$M(H_3L)$	5.4	3.57	247	$7.14 \cdot 10^7$
				2.82	261	$5.64 \cdot 10^7$
				1.78	277	$3.56 \cdot 10^7$
				0.86	352	$1.72 \cdot 10^7$
				1.40	407	$2.80 \cdot 10^7$
		$M(H_2L)$	5.8	4.01	248	$8.02 \cdot 10^7$
				2.13	277	$4.26 \cdot 10^7$
				1.67	404	$3.34 \cdot 10^7$
		ML	6.4	3.87	249	$7.74 \cdot 10^7$
				2.02	277	$4.04 \cdot 10^7$
				1.96	400	$3.92 \cdot 10^7$
		ML(OH)	7.4	3.73	249	$7.46 \cdot 10^7$
				2.00	277	$4.00 \cdot 10^7$
				2.16	400	$4.32 \cdot 10^7$
		$ML(OH)_2$	9.6	3.60	248	$7.20 \cdot 10^7$
				1.90	273	$3.80 \cdot 10^7$
				2.33	400	$4.66 \cdot 10^7$
		$ML(OH)_3$	11.0	3.57	249	$7.14 \cdot 10^7$
				1.86	273	$3.72 \cdot 10^7$
				2.33	400	$4.66 \cdot 10^7$

Table S4. Absorption data for the complexes formed in the studied systems (1:2 ratio)

Species			pH	A	λ_{max} (nm)	ε ($dm^3 \cdot mol^{-1} \cdot cm^{-1}$)
1:2	Eu(III)	$M(H_3L)_2$	5.0	1.04	241	$4.16 \cdot 10^7$
				0.78	257	$3.12 \cdot 10^7$
				0.42	280	$1.68 \cdot 10^7$
				0.26	342	$1.04 \cdot 10^7$
				0.32	417	$1.28 \cdot 10^7$
		$M(H_2L)_2$	6.1	1.34	243	$5.36 \cdot 10^7$
				0.74	260	$2.96 \cdot 10^7$
				0.49	404	$1.96 \cdot 10^7$
		$M(HL)_2$	7.0	1.60	243	$6.40 \cdot 10^7$

			0.49	275	$1.96 \cdot 10^7$
			0.70	402	$2.80 \cdot 10^7$
	ML(OH)	8.8	1.59	243	$6.36 \cdot 10^7$
			0.46	276	$1.84 \cdot 10^7$
			0.74	402	$2.96 \cdot 10^7$
	$ML(OH)_2$	10.9	1.02	244	$4.08 \cdot 10^7$
			0.41	273	$1.64 \cdot 10^7$
			0.63	402	$2.52 \cdot 10^7$
Gd(III)	$M(H_3L)$	3.7	0.68	241	$2.72 \cdot 10^7$
			0.75	261	$3.00 \cdot 10^7$
			0.16	286	$6.40 \cdot 10^6$
			0.33	344	$1.32 \cdot 10^7$
			0.09	432	$3.60 \cdot 10^6$
	$M(H_3L)_2$	5.3	0.88	241	$3.52 \cdot 10^7$
			0.69	259	$2.76 \cdot 10^7$
			0.29	344	$1.16 \cdot 10^7$
			0.25	406	$1.00 \cdot 10^7$
	$M(H_2L)_2$	6.1	1.33	240	$5.32 \cdot 10^7$
			0.56	279	$2.24 \cdot 10^7$
			0.49	414	$1.96 \cdot 10^7$
	$M(HL)_2$	6.6	1.48	243	$5.92 \cdot 10^7$
			0.44	275	$1.76 \cdot 10^7$
			0.64	404	$2.56 \cdot 10^7$
	ML(OH)	7.5	1.49	244	$5.96 \cdot 10^7$
			0.43	273	$1.72 \cdot 10^7$
			0.68	402	$2.72 \cdot 10^7$
	ML_2	8.2	1.54	243	$6.16 \cdot 10^7$
			0.46	272	$1.84 \cdot 10^7$
			0.71	404	$2.84 \cdot 10^7$
	$ML_2(OH)$	10.0	1.46	244	$5.84 \cdot 10^7$
			0.42	275	$1.68 \cdot 10^7$
			0.68	401	$2.72 \cdot 10^7$
Tb(III)	$M(H_2L)$	3.8	0.86	241	$3.44 \cdot 10^7$
			0.98	261	$3.92 \cdot 10^7$
			0.24	285	$9.60 \cdot 10^6$
			0.43	343	$1.72 \cdot 10^7$
			0.12	422	$4.80 \cdot 10^6$
	M(HL)	5.6	1.27	242	$5.08 \cdot 10^7$
			0.91	258	$3.64 \cdot 10^6$
			0.35	348	$1.40 \cdot 10^7$
			0.39	401	$1.56 \cdot 10^6$
	ML(OH)	6.6	1.90	241	$7.60 \cdot 10^7$
			0.58	274	$2.32 \cdot 10^7$
			0.83	402	$3.32 \cdot 10^7$
	ML_2	7.9	1.98	243	$7.92 \cdot 10^7$
			0.60	274	$2.40 \cdot 10^7$
			0.94	403	$3.76 \cdot 10^7$
	$ML_2(OH)$	9.1	1.95	243	$7.80 \cdot 10^7$
			0.60	274	$2.40 \cdot 10^7$
			0.90	403	$3.60 \cdot 10^7$
	$ML_2(OH)_2$	10.1	1.92	244	$7.68 \cdot 10^7$

		0.56	277	$2.24 \cdot 10^7$
		0.92	402	$3.68 \cdot 10^7$
		1.83	243	$7.32 \cdot 10^7$
$ML_2(OH)_3$	10.9	0.56	275	$2.24 \cdot 10^7$
		0.90	403	$3.60 \cdot 10^7$

Table S5. Absorption data for the ligand *N,N′*-bis(3-bromo-5-chlorosalicylidene)-2-hydroxy-1,3-propanediamine

Species	pH	A	λ_{max} (nm)	ε ($dm^3 \cdot mol^{-1} \cdot cm^{-1}$)
		2,13	245	$4.26 \cdot 10^7$
H_4L	4.5	0,86	282	$1.72 \cdot 10^7$
		0,36	351	$7.20 \cdot 10^6$
		0,98	410	$1.96 \cdot 10^7$
		2,49	246	$4.98 \cdot 10^7$
H_3L	6	0,98	277	$1.96 \cdot 10^7$
		1,29	407	$2.58 \cdot 10^7$
		2,45	242	$4.90 \cdot 10^7$
H_2L	7.2	0,85	273	$1.70 \cdot 10^7$
		0,12	323	$2.40 \cdot 10^6$
		1,27	403	$2.54 \cdot 10^7$
		2,40	246	$4.80 \cdot 10^7$
HL	9	0,84	270	$1.68 \cdot 10^7$
		0,15	318	$3.00 \cdot 10^6$
		1,25	402	$2.50 \cdot 10^7$
		2,42	244	$4.84 \cdot 10^7$
L	10.5	0,78	275	$1.56 \cdot 10^7$
		0,12	318	$2.40 \cdot 10^6$
		1,26	400	$2.52 \cdot 10^7$
